# Niche deconvolution of the glioblastoma proteome reveals a distinct infiltrative phenotype within the proneural transcriptomic subgroup

**DOI:** 10.1038/s41597-022-01716-5

**Published:** 2022-10-01

**Authors:** K. H. Brian Lam, Phedias Diamandis

**Affiliations:** 1 Department of Laboratory Medicine and Pathobiology, University of Toronto, Toronto, Ontario M5S 1A8 Canada; 2 Princess Margaret Cancer Center, University Health Network, Toronto, Ontario 610 University Avenue, M5G 2C1 Canada; 3 Department of Pathology and Laboratory Medicine, David Geffen School of Medicine, University of California Los Angeles, Los Angeles, USA; 4 Laboratory Medicine Program, University Health Network, 200 Elizabeth Street, Toronto, ON, Toronto, Ontario M5G 2C4 Canada; 5 Department of Medical Biophysics, University of Toronto, Toronto, Ontario M5S 1A8 Canada

**Keywords:** Proteomics, Machine learning, CNS cancer, Cancer microenvironment

## Abstract

Glioblastoma is often subdivided into three transcriptional subtypes (classical, proneural, mesenchymal) based on bulk RNA signatures that correlate with distinct genetic and clinical features. Potential cellular-level differences of these subgroups, such as the relative proportions of glioblastoma’s hallmark histopathologic features (e.g. brain infiltration, microvascular proliferation), may provide insight into their distinct phenotypes but are, however, not well understood. Here we leverage machine learning and reference proteomic profiles derived from micro-dissected samples of these major histomorphologic glioblastoma features to deconvolute and estimate niche proportions in an independent proteogenomically-characterized cohort. This approach revealed a strong association of the proneural transcriptional subtype with a diffusely infiltrating phenotype. Similarly, enrichment of a microvascular proliferation proteomic signature was seen within the mesenchymal subtype. This study is the first to link differences in the cellular pathology signatures and transcriptional profiles of glioblastoma, providing potential new insights into the genetic drivers and poor treatment response of specific subsets of glioblastomas.

## Introduction

Glioblastoma is an aggressive form of brain cancer that has seen little change in its clinical outlook in over 50 years^1^. While historically considered as a single and uniform disease entity, many molecular profiling efforts over the past decade have defined multiple molecular subgroups with distinct genetics, transcriptional profiles, and clinical outcomes^2–4^ Based on bulk tumor-intrinsic transcriptional signatures, glioblastoma has three major molecular subtypes known as proneural-, classical- and mesenchymal-tumors that correlate with specific genetic and clinical patterns^3^. Mesenchymal-type glioblastomas show characteristic NF1 mutations and are associated with poor clinical outcomes^5^. Glioblastoma with a classical transcriptional signature shows stereotypical EGFR and chromosome 10 alterations that often define adult-type IDH-wildtype tumors^3^. Proneural glioblastomas, associated with PDGFRA alterations, are a clinically interesting subtype as they are enriched for IDH-mutated glioblastomas^3^. Peculiarly, unlike the other two subtypes, these tumors show a relatively poor response to chemoradiation therapy that is not well understood.

Tumor histomorphology, the study of the cellular composition of tissue through microscopy, remains a powerful tool for understanding pathologies. This is especially true when there are objective and consistent histological differences between defined subgroups or classes of pathology^6^. Not only can they provide important insights into the cellular biology of various diseases, but they can also reveal potential systematic biases in large bulk-based molecular analyses. For example, originally, Verhaak and colleagues proposed four glioblastoma subtypes, which included the three aforementioned subgroups (classical, mesenchymal, proneural) and an additional “neural” class that displayed a neuronal-like pattern of expression and no characteristic mutational profiles^2^. The latter group was subsequently removed due to suspected contamination of the transcriptional signature from normal brain tissue elements that mirrored that defined by the IvyGAP resource^3,7^. Specifically, this later initiative used laser capture microdissection to carefully delineate and individually generate niche-specific transcriptome of the five major histomorphologic features of glioblastoma (cellular tumor (CT), infiltrating region (IT), microvascular proliferation (MVP), tumor cells around necrosis (PAN) and adjacent leading edge brain tissue (LE)). Such studies underscore the importance of histopathologic and molecular correlations when building disease models from large-scale bulk-profiling studies to reduce systematic biases in data generation. Furthermore, creating molecular catalogs of these morphologic features allows such comparative analysis to be done computationally and objectively by removing potential subjective interpretive variation when human observers estimate these elements.

Recently, we and others have also taken advantage of these regionally-defined public resources to align histomorphologic features to the four plastic cellular states of glioblastoma (astrocyte-like (AC, oligodendroglial progenitor-like (OPC), neural progenitor-like (NPC) and mesenchymal (MES)), as defined by single-cell RNA sequencing^8–10^. Indeed, such analyses support that certain glioblastoma single-cell phenotypes may be preferential found or influenced by their microenvironmental niches (e.g. enrichment of MES signature in PAN tumor regions). Because of the strong understanding of the biological process occurring in these regions (e.g. hypoxia within PAN regions), such morphologic-molecular correlations can further refine our cancer models and subsequent therapeutic strategies.

**Fig. 1:**
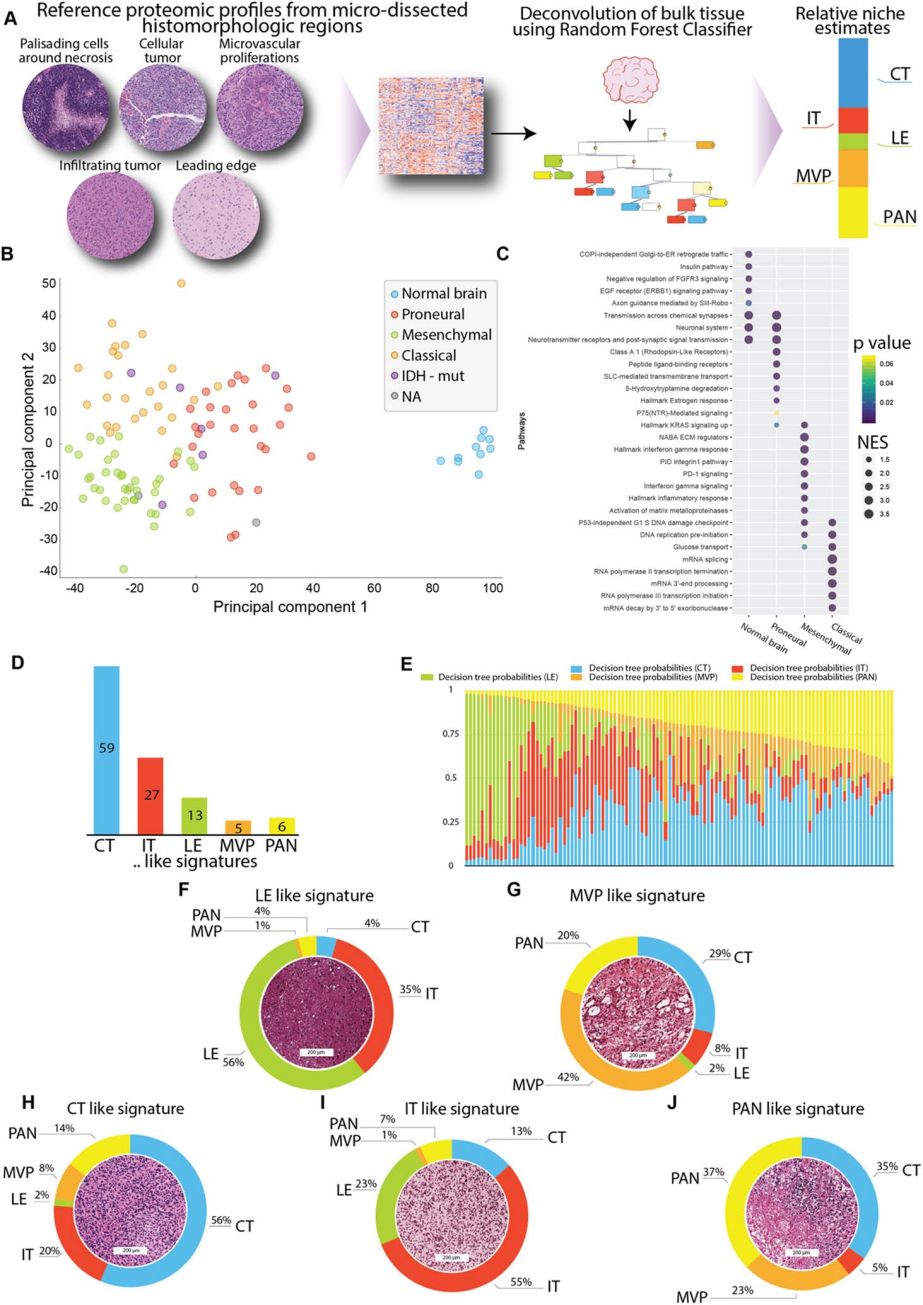
Deconvolution and estimation of histomorphological niches within bulk glioblastoma proteomes. (**A**) Schematic overview of our methodology to estimate niche proportions using reference microdissected proteomic profiles and classifying bulk tumor samples via a random forest algorithm. Hematoxylin and Eosin (H&E) images detailing the anatomical niches within GBM: leading edge (LE), infiltrating tumor (IT), cellular tumor (CT), microvascular proliferations (MVP), and palisading cells around necrosis (PAN). (**B**) Multidimensional scaling of CPTAC samples based on all proteins using principal component analysis highlights distinct grouping of TCGA subtypes (n = 110). (**C**) Gene Set Enrichment Analysis (GSEA) based on all samples and their comparisons against other sample types highlights similarities in pathways between the Normal brain samples and the proneural subgroup. Normalized enrichment score (NES) is derived from the GSEA output and accounts for differences in gene set size and in correlations between gene sets in the expression dataset. (**D**) Random forest algorithm trained on a proteomic dataset of histomorphological features classifies CPTAC proteomic samples into niche like signatures. Cases are classified into niches based on the major niche contribution. The machine learning classifications on the X-axis represent the most abundant feature. (**E**) A stacked bar chart highlights the variability of decision tree probabilities across the tumors and normal brain samples (n = 108). Machine learning classified proteomes show concordance with H&E slide images for (**F**) LE, (**G**) MVP, (**H**) CT, (**I**) IT, and (**J**) PAN -like signatures. These H & E images are representative sections and not whole slide images. Source data are provided as a Source Data file.

The manual and subjective nature of histomorphologic analysis and quantification has limited integration of this information in large molecular cohorts of glioblastoma. However, this barrier could be overcome by similar computational methods that benefit the molecular cataloging of well-defined and relevant histomorphologic features. Since many of these subtypes align with relevant clinical outcomes and genetic alternations, additional phenotypic differences could provide important unappreciated clinicopathologic correlations. Microscopically, glioblastoma shows a reliable set of “hallmark” histomorphologic features that include tumor areas of high cellularity (CT), brain infiltration (IT), microvascular proliferation (MVP) and hypoxia in which tumor cells form palisading structures around necrosis (PAN) in heterogenous quantities (Fig. 1A). As these features drive different aspects of treatment challenges in glioblastoma, defining potential relationships between different tumor niche proportions and molecular patterns could shed additional biological insights into these transcriptional classes. Towards addressing this, we recently used liquid chromatography tandem mass spectrometry (LC-MS/MS) to develop a spatially-defined proteomic atlas of glioblastoma. Specifically, we microscopically isolated and profiled the aforementioned tumor regions (CT, MVP, PAN, IT) and adjacent brain tissue from the leading edge (LE), allowing components of the glioblastoma proteome to be aligned with their relevant histomorphologic niches (n = 77 regions from 20 individual patients)^11,12^. Moreover, because protein patterns provide a reliable downstream phenotypic readout of biological function, these regional profiles could also serve as valuable reference sets to estimate niche contributions of bulk tissue-derived profiles.

Such deconvolution techniques have already been highly successful at the cellular level, where cell-type enriched transcriptomes/proteomes are used to computationally estimate the proportions of different cell types within bulk-profiled tissue. These cellular decomposition methods are important as they can leverage the high-throughput nature of large-scale bulk-profiling studies while providing important cell-type proportions that often require more sophisticated and low-throughput single-cell analyses. Integrating molecular profiles with information about tissue heterogeneity has provided important insights into relationships between cell type composition and diseases. They also overcome the confounding effects of tissue composition when drawing conclusions from these large-scale studies^13–20^. Here, we extend this concept by using the histologically-defined proteomes of hallmark glioblastoma features to carry out”niche deconvolution” of a large proteogenomically characterized glioblastoma cohort (n = 110) from the Clinical Proteomic Tumor Analysis Consortium (CPTAC)^21^. We compare the resulting estimates of the relative niche contributions across the distinct transcriptional subtypes to garner potential new biological and clinical insights into this aggressive disease.

## Results

### Estimation of histomorphologic features in bulk glioblastoma proteomes

**Fig. 2:**
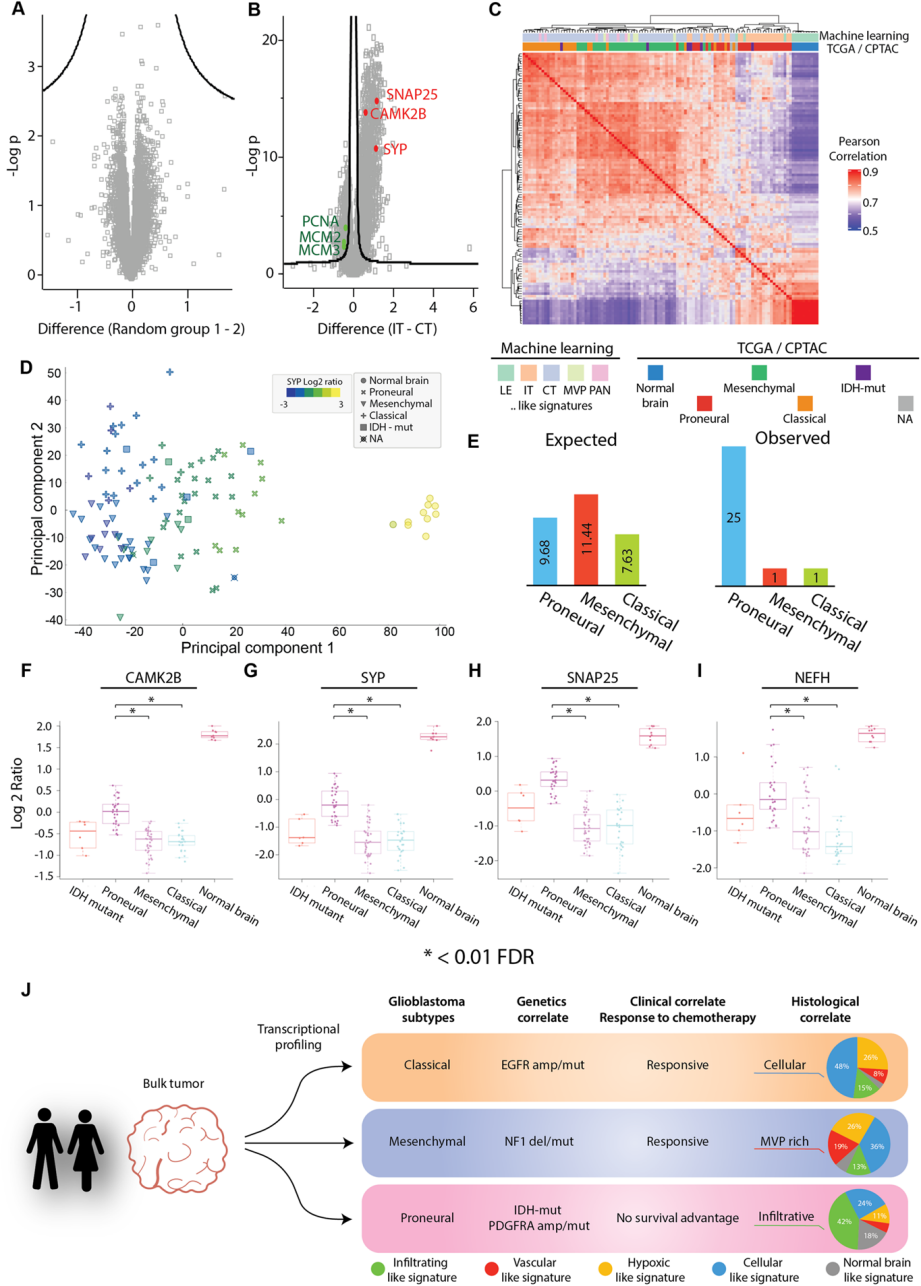
Association of the proneural subgroup with the infiltrating tumor phenotype. Differentially enriched protein (DEP) analysis by volcano plot comparing (**A**) randomized groupings of tumor samples (n = 86, randomly distributed into two groups of 43) and (**B**) machine learning classified tumor samples highlights distinct phenotypic tumors (n = 86, FDR 0.05, S0 > 0.1). (**C**) Unsupervised hierarchical clustering of CPTAC proteomic samples by Pearson correlation utilizing all proteins highlights an association between the infiltrative-like signature and the proneural subgroup (n = 110). (**D**) Multidimensional scaling of CPTAC samples based on all proteins using principal component analysis highlights increasing abundances of the synaptic marker SYN from left to right (n = 110). (**E**) Distribution of expected and observed abundances of IT-like signature tumor samples based on the total number of samples identified as IT-like signature tumors by the random forest classifier. (**F**) Comparison of CAMK2B by boxplot highlights enrichment within the proneural subgroup against other tumor subtypes; proneural vs mesenchymal (FDR = 3.97e-12), proneural vs classical (FDR = 5.18e-11), proneural vs IDH mutant (FDR = 0.86). (**G**) Comparison of SYP by boxplot highlights enrichment within the proneural subgroup against other tumor subtypes; proneural vs mesenchymal (FDR = 1.30e-12), proneural to classical (FDR = 4.15e-11), proneural to IDH mutant (FDR = 1). (**H**) Comparison of SNAP25 by boxplot highlights enrichment within the proneural subgroup against other tumor subtypes; proneural vs mesenchymal (FDR = 8.51e-18), proneural vs classical (FDR = 8.01e-12), proneural vs IDH mutant (FDR = 0.36). (**I**) Comparison of NEFH by boxplot highlights enrichment within the proneural subgroup against other tumor subtypes; proneural vs mesenchymal (FDR = 7.71e-5), proneural vs classical (FDR = 9.56e-8), proneural vs IDH mutant (FDR = 1). Data are presented as median values +/− IQR and min/max values (whiskers). P values were first calculated based on proteins from all samples utilizing a one-tailed t-test and then adjusted for the Benjamini-Hochberg correction (n = 108). (**J**) Schematic summary of the various features of the TCGA GBM subgroups, including genetics, clinical and histomorphologic correlates. Source data are provided as a Source Data file.

Visualization of the LC-MS/MS protein dataset of the CPTAC glioblastoma cohort through principal component analysis showed that proteomic programs were faithfully segregated with transcriptional subtypes (Fig. 1B). We next used gene set enrichment analysis (GSEA) of the proteomic data to explore protein-level programs of each transcriptional subtype. In addition to showing distinct protein-level programs within these subgroups, this analysis highlighted a possible enrichment of contaminating normal neural tissue within the proneural subgroup (Neuronal Systems, fold enrichment = 3.63, FDR < 0.0001; Fig. 1C). To more formally explore this, we next used reference niche-enriched proteomic profiles from a recent study (Fig. S1)^11,12^ to train a random forest classifier that would use regional protein-based patterns to estimate the relative contributions of the different histomorphologic niches (CT, MVP, IT, LE, PAN) within bulk-profiled samples of the CTPAC cohort (Fig. 1A,). Performance testing of this model using a random sampling method of the 154 samples derived from different tissue niches across the 20 patient samples show strong classification performance (area under the receiver operator curve (AUC) = 0.997, Fig. S2). Classification of the CTPAC samples using this model highlighted significant heterogeneity of the estimated niches contribution across samples (Fig. 1D,E, Table S1). Reassuringly, examination of available whole slide images often showed concordance of histomorphologic features with this protein-based niche estimates, as seen in the representative sections in Fig. 1F–J. These estimates were also validated by confirming concordant differences of classic individual markers of hypoxic (carbonic anhydrase IX, CAIX), vascular (CD34), and neuronal (Synaptosomal-Associated Protein, 25 kDa, SNAP25) tissue (Supplementary Fig. 3). Together, this suggests that despite best efforts of many bulk-based profiling studies to select samples enriched in cellular tumor regions, expression-based molecular patterns may contain substantial heterogeneous contributions of non-CT niches regions, underscoring the importance of niche deconvolution to control the sensitivity and specificity of such analyses. For example, while very few differences are identified when the cohort is randomized into 2 groups for comparison, substantial protein enrichment of the same samples can be recovered why stratifying based on their predominating niche-specific proteomic signature predicted by our model (FDR = 0.05, S_0_ > 0.1 Fig. 2A,B). This suggests a possible systematic bias of proteomes following niche deconvolution.

### Aligning the TCGA subtypes with histomorphological features

Given that current transcriptional subtypes of glioblastoma are generated using “tumor-intrinsic signatures”, we next explored relationships with our protein-based niche-estimates. Interestingly, unsupervised Pearson correlation of the proteomic data organized by transcriptional subtypes showed distinct protein-level signatures, including the possible enrichment of infiltrating tumor-like niches within the proneural subgroup (Fig. 2C). To validate this observation, we first explored the abundance of synaptophysin (SYP), a classic marker of normal neuronal tissue on the PCA plot. Indeed, samples enriched for SYP were positioned closer to the normal brain control samples (Fig. 2D). Chi-squared testing of the distribution of IT-like samples in the proneural glioblastomas in the CPTAC data also confirmed this relationship (χ^2^ test p = 0.001936, Fig. 2E). We again support and confirm these deconvolution estimates with individual well-characterized protein markers across the different transcriptional subgroups. Within the proneural subgroup, we observed a significant elevation of neuronal proteins at the dendrite (calcium/calmodulin dependant protein kinase, CAMK2B, Fig. 2F, FDR < 0.01), synaptic terminus-level (SYN, Fig. 2G, FDR < 0.01), axonal (SNAP25, Fig. 2H, FDR < 0.01), and axoskeleton (neurofilament heavy chain, NEFH, Fig. 2I, FDR < 0.01). Interestingly, in addition to the large contribution of an infiltrative compartments within proneural tumors, we also observed relatively lower estimates of microvascular proliferation (p = 2.59 × 10^−8^ vs. mesenchymal) and hypoxic (p = 1.28 × 10^−15^ vs. mesenchymal) niches within this subtype (Fig. S4). Together these findings support a potential predilection for a gliomatosis-like growth pattern^22^ of proneural glioblastoma with a tendency to diffusely invade brain tissue (IT-like niche) preferentially compared to a mass forming cellular tumor-like pattern (CT-like pattern) and other WHO grade 4 histologic features (MVP/PAN). Similarly, this analysis suggests that the mesenchymal transcriptional subtype may be enriched for WHO grade 4 histomorphologies (e.g. PAN-like, MVP-like molecular signatures) as compared to the other classes.

## Discussion

Virchow’s concept that cytoarchitectural and molecular aberrations in tissue can predict clinical manifestations of the disease is the fundamental cornerstone of modern medicine. Even during this era of molecularly focused, phenotypic differences in tissue organization remain vital in garnering mechanistic insight into specific genetic drivers and their downstream clinical consequences. However, their relative subjectivity and heterogeneity of histomorphology across tissue samples have challenged their utility in modern large-scale molecular profiling initiatives. Because of the central position along the clinical pathologic spectrum, systematic quantification may nonetheless still aid in solidifying missing connections and improving our models of cancer and other diseases.

Here we leveraged histomorphologically-defined proteomic signatures and machine learning to deconvolute and estimate niche proportion differences in a proteogenomic cohort of glioblastoma. In addition to helping triage tumor samples based on specific niches they may best represent, we uncover a strong association of the transcriptional glioblastoma subtypes with histomorphologic hallmarks (Fig. 2J). The large number of IT-dominated samples allowed us to make a particularly strong connection between proneural glioblastomas and a diffusely infiltrating phenotype. This finding could help unify some unexplained clinicopathologic correlations associated with this subset of tumors. For example, a recent study of pediatric gliomatosis cerebri, an aggressive and highly infiltrative form of glioma, classified as either IDH-mutated (17%) or RTK I (PDGFRA) (44%) by methylation profiling^23^; molecular features associated with the proneural subgroup. Interestingly, some experimental models also support a pro-infiltrative role of PDGFRA in glioma stem cells^24^, the transcriptional subtype-genetic-phenotype association proposed by our analysis. The invasion-promoting effects of PDGF is also supported in other non-nervous system tumor types and argues that PDGFRA signaling in glioma may serve more than just a growth factor/proliferation signal^25–27^. Moreover, the relative paucity of permeable tumor-derived vasculature and associated hypoxic/necrotic-like regions found in our analysis of proneural tumors may also help partially explain the relatively poor response to chemotherapy of this tumor subtype.

There are some important caveats of our study that are worth considering. It is possible that tissue sent for expression-based profiling (e.g. proteomics/transcriptomics) may be enriched for certain microenvironmental regions (infiltrating edge, hypoxia) simply due to random sampling. While we believe this could partially explain some of the sample-to-sample variations we identified, it likely cannot fully account for the reported observations. For example, initial studies describing the transcriptional subtypes of glioblastoma proposed four distinct subgroups; proneural, mesenchymal, classical and an additional “neural” class. This neural subgroup had an expression profile very similar to leading edge brain tissue and a paucity of identifiable mutations. In this case, this lack of clinical and genetic correlates made this subgroup less informative and suspected to be partially derived from random oversampling of primarily adjacent non-neoplastic brain tissue. Similarly, unavoidable niche contaminations likely plague the genuine gene expression signature of the other subgroup, including vascular and necrotic regions within the mesenchymal glioblastoma subtype^3^. This is a known limitation of bulk profiling initiatives and is difficult to remove with computational approaches entirely. Perhaps what distinguishes the former three subtypes discussed in this paper from the limitations that plagued the neural subclass is their non-random distribution of mutations and clinical characteristics. The enrichment of specific mutations (e.g. PDGFRA) in the proneural subgroup serves as a critical ground truth and links the potential contaminating brain tissue as an inherent biological consequence associated with the genetic events characteristic of this subgroup.

Similarly, because of the bulk profiling nature of our analysis, we also cannot rule out the possibility that these “candidate” neuronal genes are not also intrinsically expressed by tumoral cells. We find this alternative hypothesis, as least in the sense that the overwhelming neuronal signal is coming from neoplastic cells, less likely. Firstly, both low-grade diffusely infiltrating gliomas and gliomas displaying the “gliomatosis” pattern, and commonly classify as “proneural”, often lack a definitive mass on imaging and histologically often show a definitive inter-mixed histomorphology on both H&E and immunohistochemical analysis. Moreover, the expression of classical neuronal genes spanning the axonal (NFH) and synaptic (SYN) compartment by glial cells also goes against clinical wisdom and experience. In some cases, these neuronal building blocks were found at >30–50% the levels seen in control brain tissue parenchyma. While we of cannot completely rule out this alternative explanation, we believe contamination of infiltrated brain tissue is the more conservative interpretation based on current knowledge. These observations should however be further validated in large tissue cohorts with careful immunohistochemical localization of such neuronal proteins in glioblastomas belonging to the proneural subtype.

Finally, similar to the popularity of cell-type decomposition approaches, we believe this proteohistomoic deconvolution approach can be adapted to other histomorphologically-defined features and cancer types. This could aid in the development of more advanced tumor models that eliminate bias and incorporate potentially critical morphologic correlates to clinically significant drug response, outcomes and genetic events.

## Methods

### Ethics statement

Datasets used in this study were retrieved from previously published, anonymized, publicly available resources; thus, additional institutional research ethics board approval was not applicable.

### Development of histomorphologic signatures of glioblastoma

Previously, we leveraged mass spectrometry-based proteomics to develop a human glioblastoma atlas that aligns proteomic patterns to hallmark histomorphological features and highlights niche-specific phenotype-level biomarkers and targets^11^. The cohort consisted of 20 patients with multiple classical features, where selection of anatomical niches was standardized by independent annotations provided from two board-certified neuropathologists. TME differences across patient samples were normalized using the same selection criteria as IvyGAP^7^. Leading Edge (LE) is the outermost boundary of the tumor, and should represent intrinsically normal brain-like signatures. Infiltrating Tumor (IT) is the intermediate zone between the Leading Edge (LE) and Cellular Tumor (CT). Cellular Tumor (CT) comprises the central part of the tumor core. Pseudopalisading Cells around Necrosis (PAN) is the narrow boundary of cells arranged like pseudopalisades along the perimeter of necrosis. Microvascular Proliferation (MVP) refers to two or more blood vessels sharing a common vessel wall. In total, we were able to collect 78 samples which were used to generate a total of 156 mass spectrometry duplicates. We identified 4,794 proteins across samples.

### Bioinformatic analysis

Data used in this publication were generated by the Clinical Proteomic Tumor Analysis Consortium (NCI/NIH). The training set of mass spectrometry proteomics data were downloaded from the ProteomeXchange Consortium via the PRIDE partner repository with the dataset identifier PXD019381. Data analysis was performed using a variety of biostatistical platforms Perseus 1.6.15.0 (www.coxdocs.org), R (www.r-project.org) (version 4.0.4), Orange3 python package (https://orange.biolab.si/) (version 3.31.0), and GSEA 4.1.0 (https://www.gsea-msigdb.org/gsea/index.jsp). Gene set enrichment analysis (GSEA) was used to define pathways enriched in each grouping. Heatmaps and clustering were performed using the R package ComplexHeatmap (http://bioconductor.org/packages/release/bioc/html/ComplexHeatmap.html) (version 2.9.3). Proteogenomic analysis was performed by filtering for genes only present in both datasets and averaging those values across samples in a grouping and then performing spearman rank correlation. Volcano plots were generated using Perseus with (FDR = 0.05, S_0_ > 0.1).

### Preprocessing of proteomics data

Mass spectrometry raw data files were processed using MaxQuant Andromeda (version 1.5.5.1) search engine (www.coxdocs.org) against the Human Swissprot protein database (July, 2019 version). The mass spectrometry proteomics data have been deposited to the ProteomeXchange Consortium via the PRIDE partner repository with the dataset identifier PXD019381. Processing of proteomic data was performed using biostatistical platforms Perseus (www.coxdocs.org). Samples were annotated according to their anatomical groups. Proteins were filtered such that only those that appeared in at least 60% within a group were included. The raw values were Log2 transformed and non-valid values were imputed (downshift = 0.3, width = 1.8). The distribution of proteomic values were assessed via histograms for a normal distribution.

### Random forest classifier

Random forest classifier was trained on 154 laser capture micro dissected regions from an anatomical proteomic data set^11^ [https://www.brainproteinatlas.org/dash/apps/GPA] using the Orange3 python package (v 3.31.0). The model included 200 decision trees and unlimited depth until 95% of classes are accurately classified. Random sampling was performed such that 80% of samples within each grouping were used for training while the remaining 20% were used for testing. The results were assessed by confusion matrix and ROC analysis. This machine learning classifier was then applied to the 110 samples from the CPTAC GBM discovery cohort. Data used in this publication were generated by the Clinical Proteomic Tumor Analysis Consortium (NCI/NIH) and are publicly available [https://cptac-data-portal.georgetown.edu/study-summary/S048]. For classification across data sets proteomic values were normalized within patient samples by z-score. Normalization was performed to interrogate patterns in molecular signatures and not as a comparison between LFQ and TMT quantifications strategies. All other analysis used raw values. The classifications and decision tree probabilities can be accessed within Table S1. Decision tree probabilities were used to estimate abundances of each anatomical niche with the greatest abundance being assigned as the “… like signature”.

## Supplementary information

Supplementary information

## Data Availability

The anatomical atlas of human glioblastoma mass spectrometry proteomics data have been deposited to the ProteomeXchange Consortium via the PRIDE^28^ partner repository with the dataset identifier PXD019381^29^. Data used in this publication were generated by the Clinical Proteomic Tumor Analysis Consortium (NCI/NIH) and are publicly available [https://cptac-data-portal.georgetown.edu/study-summary/S048]. Source data are provided with this paper.

## Code availability

All analyses were generated with existing packages, and no original code was created. The packages associated with this analysis can be found below. Data analysis was performed using a variety of biostatistical platforms Perseus 1.6.15.0 (www.coxdocs.org), R (www.r-project.org) (version 4.0.4), Orange3 python package (https://orange.biolab.si/) (version 3.31.0), and GSEA 4.1.0 (https://www.gsea-msigdb.org/gsea/index.jsp). Gene set enrichment analysis (GSEA) was used to define pathways enriched in each grouping. Heatmaps and clustering were performed using the R package ComplexHeatmap (http://bioconductor.org/packages/release/bioc/html/ComplexHeatmap.html) (version 2.9.3).
