# Supplementary material for: Niche deconvolution of the glioblastoma proteome reveals a distinct infiltrative phenotype within the proneural transcriptomic subgroup
Source: Sci Data. 2022 Oct 1;9:596. doi: 10.1038/s41597-022-01716-5 (PMC9526702; doi:10.1038/s41597-022-01716-5)
Supplement: Supplementary file 1 — Supplementary information [file 41597_2022_1716_MOESM1_ESM.pdf]

Supplemental Materials

Niche deconvolution of the glioblastoma proteome reveals a distinct infiltrative phenotype within the proneural transcriptomic subgroup

Authors: K. H. Brian Lam, Phedias Diamandis

Corresponding author: Phedias Diamandis (p.diamandis@mail.utoronto.ca)

Figure List

Figure S1. Machine learning niche specific proteomic training set

Figure S2. Random forest model classifies niche specific signatures

Figures S3. Random forest classification of bulk CPTAC samples is associated with niche specific signatures.

Figure S4. Niche deconvolution of proteomic samples by decision tree probabilities.

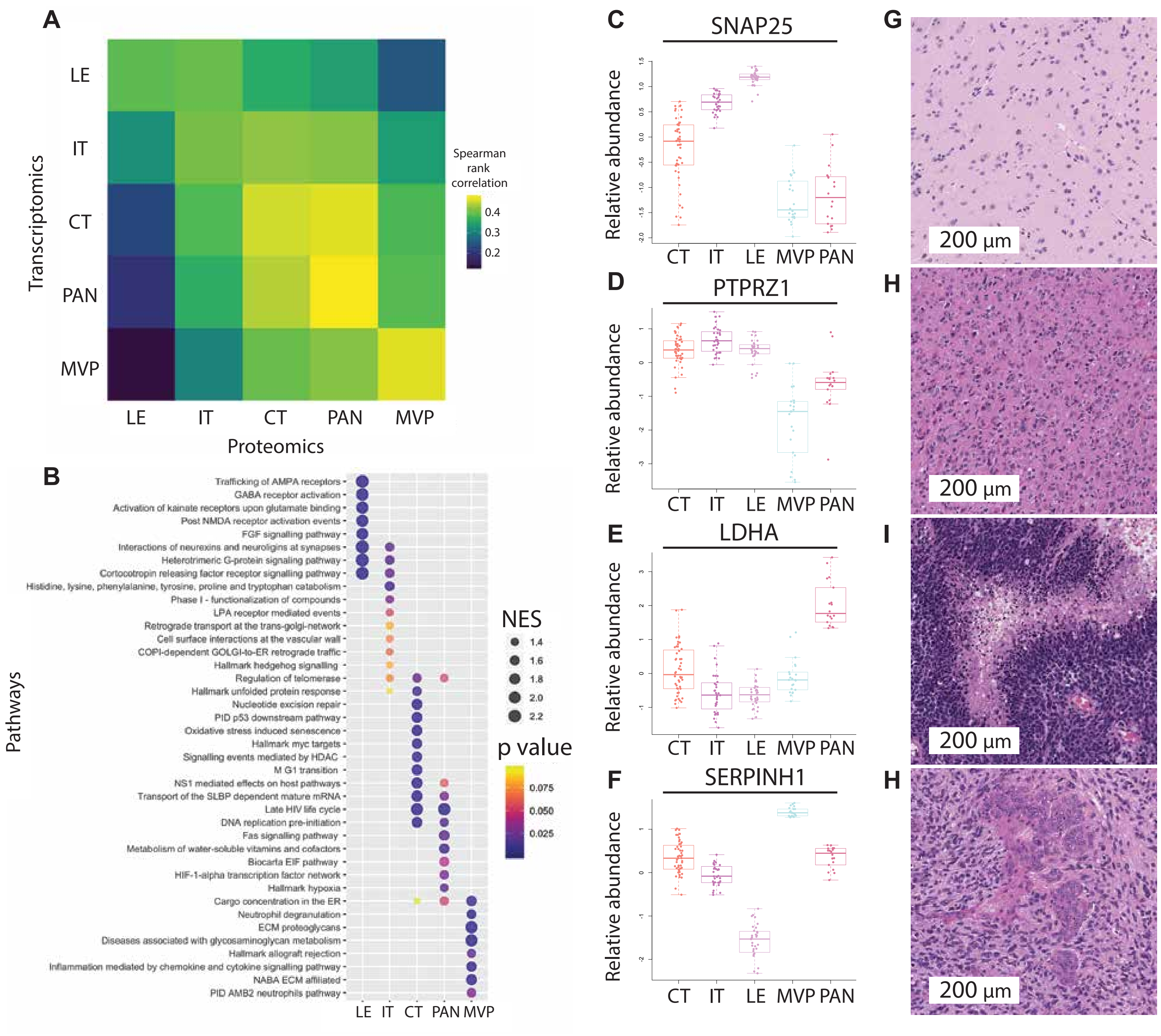

Figure S1. Machine learning niche specific proteomic training set. (**A**) Spearman rank correlation between anatomical signatures from transcriptomics (IvyGAP) and proteomics. (**B**) Gene set enrichment analysis (GSEA) based on proteomic signatures and their comparisons against other anatomical signatures. Normalized enirhcment score (NES) is derived from the GSEA output and accounts for differences in gene set size and in correlations between gene sets. Enrichment of anatomical markers (**C**) SNAP25 within LE, (**D**) PTPRZ1 within IT, (**E**) LDHA within PAN and (**F**) SERPINH1 within MVP (n=154). Data are presented as median values +/- IQR and min/max values (whiskers). H & E images of anatomical regions profiled to generate molecular signatures (**G**) LE, (**H**) IT, (**I**) PAN and (**J**) MVP.

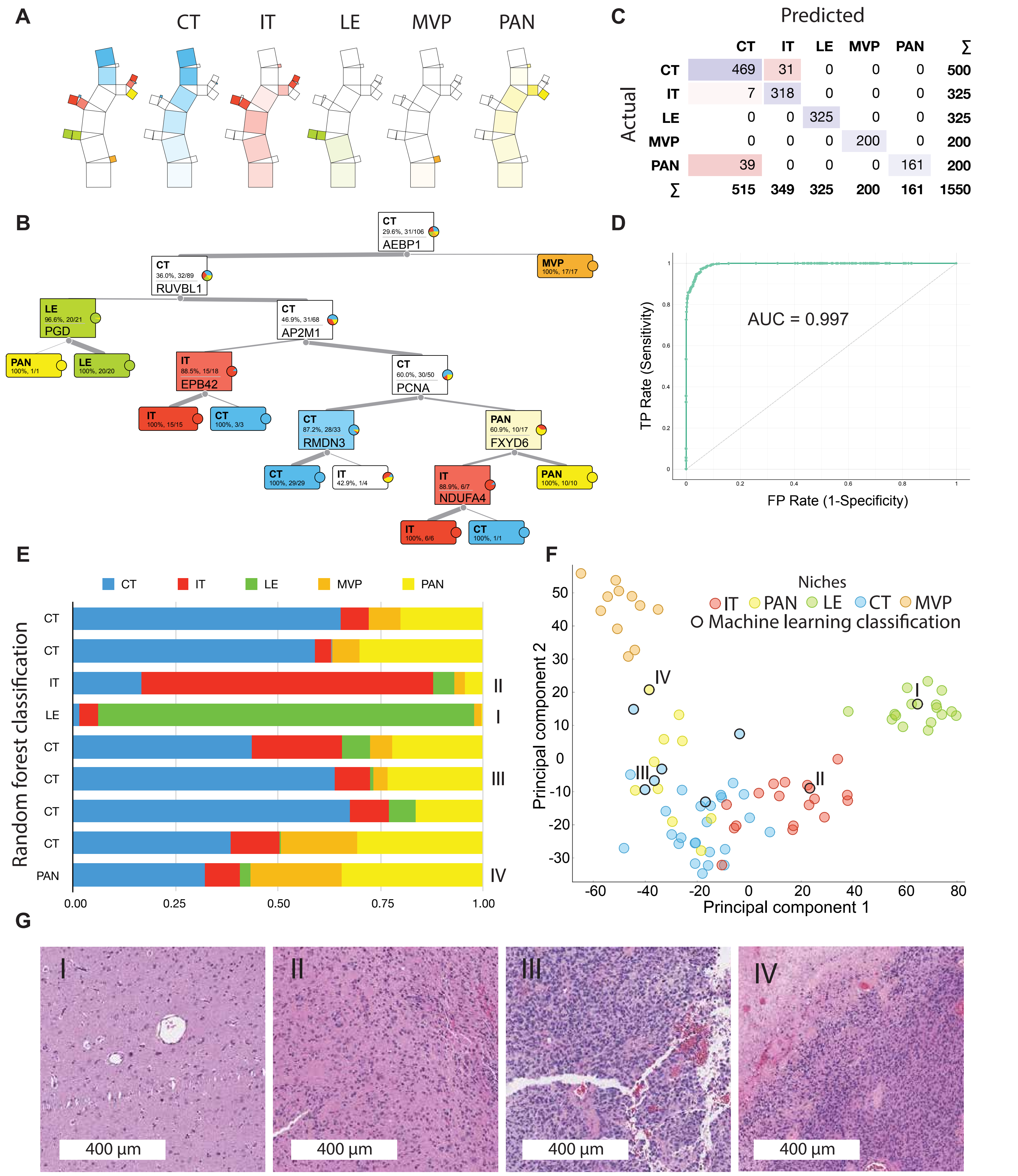

Figure S2. Random forest model classifies niche specific signatures. (**A**) Pythagoras trees depict general decision tree hierarchies highlighting the depth of analysis required for classification of proteomic samples. (**B**) A decision tree within the random forest model highlights the number of proteins used to separate classes. Random sampling was then used to evaluate the generalizability and robustness of the random forest model, (**C**) a confusion matrix indicates only 77/1550 misclassifications and (**D**) a ROC curve with AUC of 0.997. (**E**) The random forest model was then applied to 9 bulk samples to deconvolute the abundances of the five anatomical niches and identify the most abundant niche. (**F**) Multidimensional scaling of all anatomical samples and bulk samples by principal component analysis highlights random forest classification clustering with anatomical samples (n=77). (**G**) H & E images of bulk samples suggest histomorphological association of random forest classifications.

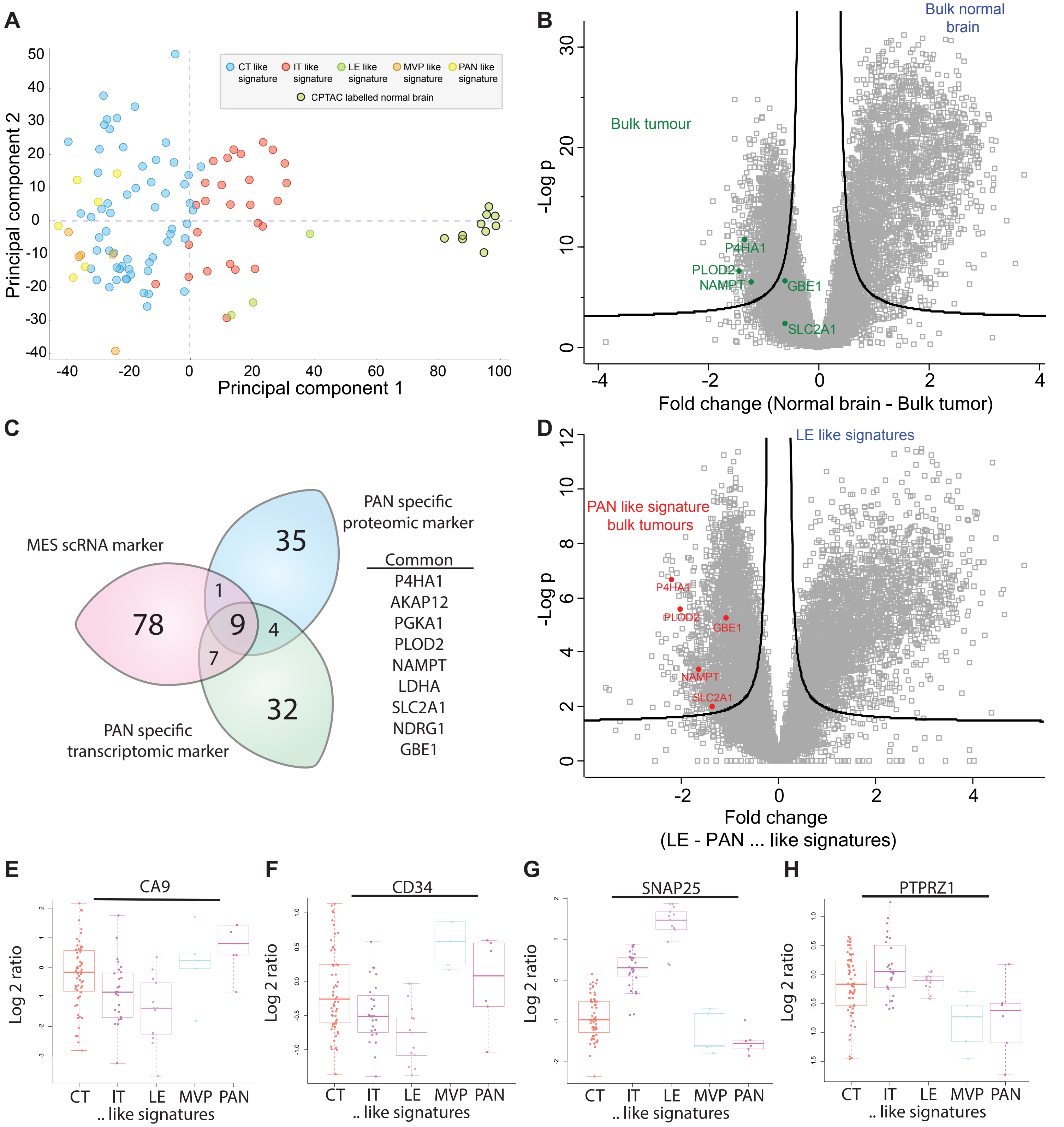

Figure S3. Random forest classification of bulk CPTAC samples is associated with niche specific signatures. (**A**) Multidimensional scaling of CPTAC samples using all proteins by principal component analysis highlight the correct classification of CPTAC labelled normal brain samples as LE like signature samples (n=110). (**B**) Venn diagram highlighting PAN specific markers from across proteomic, transcriptomic and single cell analyses from Lam *et al.*, Puchalski *et al.*, Neftel *et al.*, and Eberhart *et al.* (**C**) Volcano plot comparing normal brain and bulk tumor samples is unable to highlight PAN specific markers as statistically significant (FDR 0.05, S0 >0.1, n=110). (**D**) Volcano plot comparing PAN like signature tumors with LE like signatures highlights PAN markers as statistically significant, indicating the appropriate niche specific association (FDR 0.05, S0 >0.1, n=19). PAN specific markers from (**B**) that were not statistically significant in either (**C**) or (**D**) were not used. (**E**) Relative expression of the hypoxia marker, CA9 by boxplot highlights enrichment within the PAN like signature subgroup. (**F**) Relative expression of the endothelial marker, CD34 by boxplot highlights enrichment within the MVP like signature subgroup. (**G**) Relative expression of the neuronal marker, SNAP25 by boxplot highlights enrichment within the LE like signature subgroup. (**H**) Relative expression of the stemness and GBM invasion marker PTPRZ1 by boxplot highlights enrichment within the IT like signature subgroup. Data are presented as median values +/- IQR and min/max values (whiskers) (n=108).

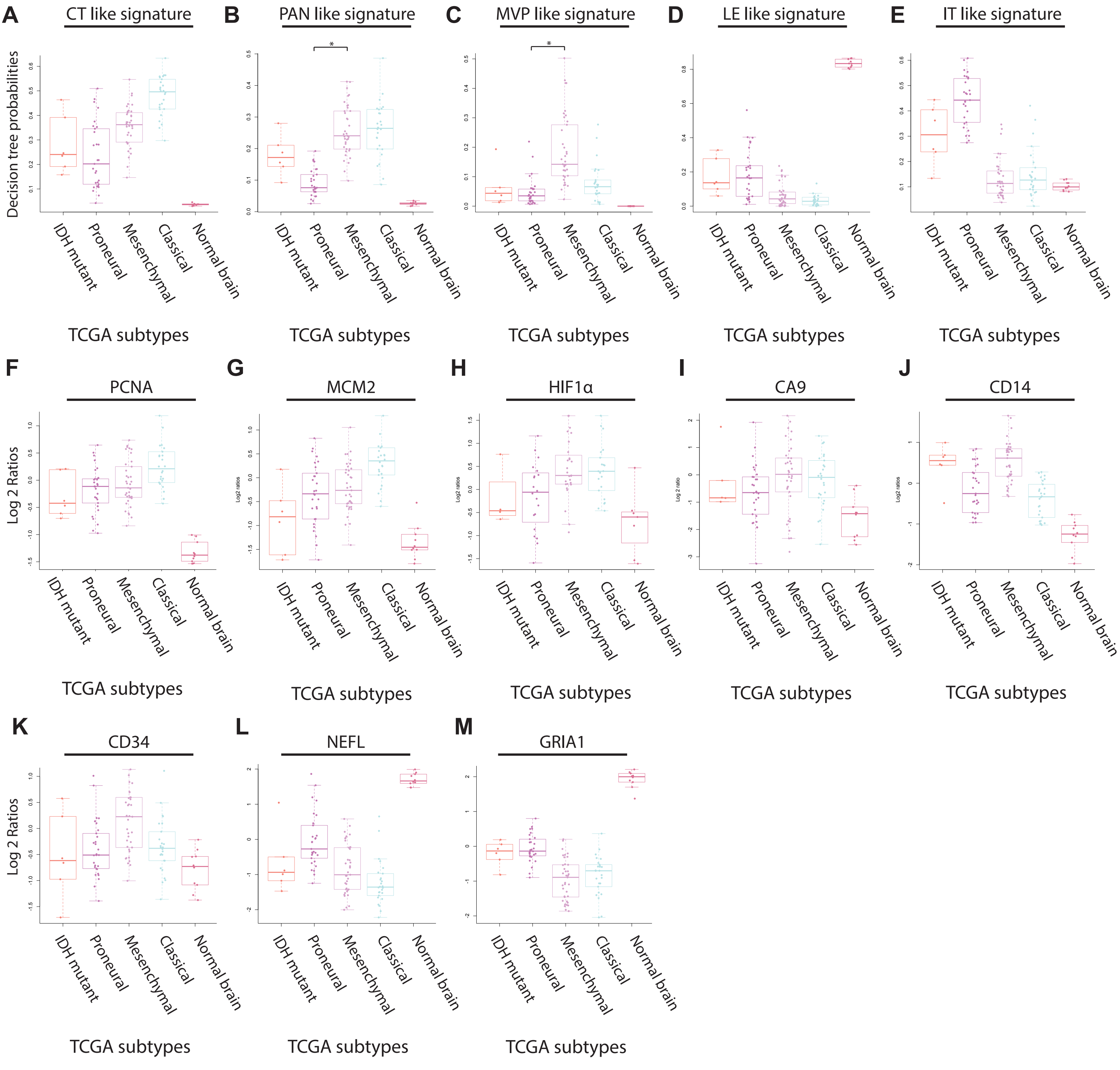

Figure S4. Niche deconvolution of proteomic samples by decision tree probabilities.
Decision tree probabilities of the (**A**) CT like signature classification, (**B**) PAN like signature, (**C**) MVP like signature (**D**) LE like signature, and (**E**) IT like signature by the random forest classifier across the different TCGA subtypes from the CPTAC tumors. Together this suggests decreased vasculature and cellularity across the proneural subgroup. MVP like signature, proneural vs mesenchymal (p = 2.59 e-8) PAN like signature, proneural vs mesenchymal (p = 1.28 e-15), (* < 0.01 p-value) p-values are calculated based on two-sided t-test (n=108). Comparison of the proliferation marker, (**F**) PCNA, and (**G**) MCM2 amongst the tumor subtypes by boxplot highlights enrichment within the Classical subgroup. Comparsion of the hypoxia marker, (**H**) HIF1α, and (**I**) CA9 by boxplot highlights decreased hypoxia in Proneural tumors. Comparsion of the endothelial marker, (**J**) CD14, and (**K**) CD34 by boxplot highlights decreased vasculature in the Proneural subgroup and enrichment in the Mesenchymal subgroup. Relative expression of the neuronal marker, (**L**) NEFL, and (**M**) GRIA1 by boxplot highlights enrichment within the Normal brain and proneural samples. Data are presented as median values +/- IQR and min/max values (whiskers) (n=108).
